# *Pyemotes ventricosus* Dermatitis: A Serpiginous Skin Lesion due to a Mite that Parasitizes a Wood-Boring Beetle

**DOI:** 10.4269/ajtmh.18-1006

**Published:** 2019-05

**Authors:** Andreas Neumayr, Esther Kuenzli

**Affiliations:** 1 Swiss Tropical and Public Health Institute, Basel, Switzerland;; 2 University of Basel, Basel, Switzerland

**Figure 1.**
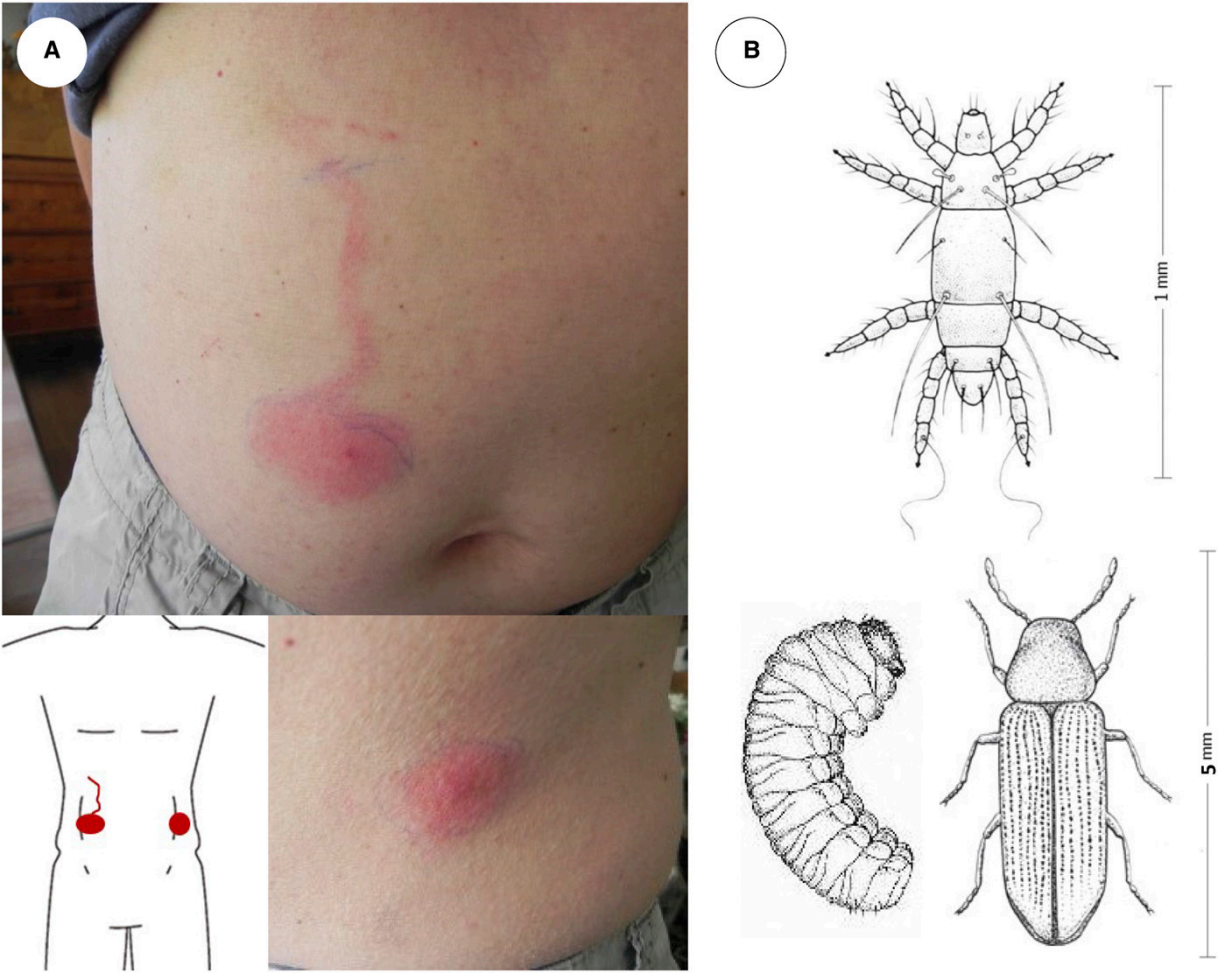
(**A**) skin lesions of the patient; (**B**) top: *Pyemotes ventricosus* mite; bottom: *Anobium punctatum* larva (left) and beetle (right). This figure appears in color at www.ajtmh.org.

In July (midsummer) 2018, a 51-year-old man residing in a village in the Swiss canton of Valais developed two highly pruritic skin lesions. One large lesion was located on the abdomen and a smaller one on the left flank (Figure 1A). Erythematous macules evolved from initial red spots to full size (pictured) within 24 hours. An erythematous serpiginous track extending cranially from the larger lesion developed in the subsequent 12 hours. Both lesions appeared shortly after he handled substantial amounts of logs and timber outdoors. Based on the lesions’ central morphology, rapid evolution, and itchiness, the consulted family physician discarded the differential diagnoses of erysipelas with lymphangitis, erythema migrans due to Lyme disease, and cutaneous larva migrans and suspected an unusual arthropod bite or sting. He referred the patient to the medical department of the Swiss Tropical and Public Health Institute for advice, where the spot diagnosis of *Pyemotes ventricosus* dermatitis was made. Under topical corticosteroid and oral antihistamine treatment, the lesions slowly regressed and finally disappeared.

*Pyemotes ventricosus* dermatitis is caused by the bite of *P. ventricosus* mites (“European straw itch mites”), free-living ectoparasites parasitizing the larvae of *Anobium punctatum* (“common furniture beetle”), a wood-boring beetle (Figure 1B).^1^ *Pyemotes ventricosus* mites occasionally infest humans handling affected wood or furniture as incidental hosts. Patients present with highly pruritic lesions consisting of a singular central vesicle/urticarial lesion (the mite’s bite site) surrounded by a rapidly expanding erythematous macule. The clinical differentiation to other arthropod bite reactions can be made if the characteristic linear or serpiginous erythematous track, called “comet sign”, is present.^2^ The lesions evolve within 24 hours after contact with *P. ventricosus*, are self-limited, and typically resolve in 1–3 weeks. Patients may also develop systemic symptoms, such as fever, chills, vomiting, or intense headache. The diagnosis can be challenging to make because the mites are not visible to the naked eye and its bites are painless.
